# Cofactor Selectivity in Methylmalonyl Coenzyme A Mutase, a Model Cobamide-Dependent Enzyme

**DOI:** 10.1128/mBio.01303-19

**Published:** 2019-09-24

**Authors:** Olga M. Sokolovskaya, Kenny C. Mok, Jong Duk Park, Jennifer L. A. Tran, Kathryn A. Quanstrom, Michiko E. Taga

**Affiliations:** a Department of Plant & Microbial Biology, University of California Berkeley, Berkeley, California, USA; b Department of Chemistry, University of California Berkeley, Berkeley, California, USA; University of California, Irvine

**Keywords:** *Sinorhizobium meliloti*, chemical diversity, cobalamin, cobamides, enzyme selectivity

## Abstract

Cobamides, a uniquely diverse family of enzyme cofactors related to vitamin B_12_, are produced exclusively by bacteria and archaea but used in all domains of life. While it is widely accepted that cobamide-dependent organisms require specific cobamides for their metabolism, the biochemical mechanisms that make cobamides functionally distinct are largely unknown. Here, we examine the effects of cobamide structural variation on a model cobamide-dependent enzyme, methylmalonyl coenzyme A (CoA) mutase (MCM). The *in vitro* binding affinity of MCM for cobamides can be dramatically influenced by small changes in the structure of the lower ligand of the cobamide, and binding selectivity differs between bacterial orthologs of MCM. In contrast, variations in the lower ligand have minor effects on MCM catalysis. Bacterial growth assays demonstrate that cobamide requirements of MCM *in vitro* largely correlate with *in vivo* cobamide dependence. This result underscores the importance of enzyme selectivity in the cobamide-dependent physiology of bacteria.

## Abstract (precis)

Cobamides, including vitamin B_12_, are enzyme cofactors used by organisms in all domains of life. Cobamides are structurally diverse, and microbial growth and metabolism vary based on cobamide structure. Understanding cobamide preference in microorganisms is important given that cobamides are widely used and appear to mediate microbial interactions in host-associated and aquatic environments. Until now, the biochemical basis for cobamide preferences was largely unknown. In this study, we analyzed the effects of the structural diversity of cobamides on a model cobamide-dependent enzyme, methylmalonyl-CoA mutase (MCM). We found that very small changes in cobamide structure could dramatically affect the binding affinity of cobamides to MCM. Strikingly, cobamide-dependent growth of a model bacterium, Sinorhizobium meliloti, largely correlated with the cofactor binding selectivity of S. meliloti MCM, emphasizing the importance of cobamide-dependent enzyme selectivity in bacterial growth and cobamide-mediated microbial interactions.

## INTRODUCTION

Cobalamin, commonly referred to as vitamin B_12_, is a versatile enzyme cofactor used by organisms in all domains of life. In humans, cobalamin is essential for methionine synthesis and the breakdown of fatty acids, amino acids, and cholesterol (1, 2). Bacteria and archaea additionally use cobalamin and related cofactors, cobamides, for deoxyribonucleotide synthesis (3), metabolism of various carbon and energy sources (4–17), synthesis of secondary metabolites (18–25), sensing light (26), and other processes (15–17, 27–32). The finding that 86% of bacterial species encode at least one cobamide-dependent enzyme in their genome (33) demonstrates the prevalence of cobamide-dependent metabolisms. Widespread use of these cofactors can be attributed to their chemical versatility, as they facilitate challenging chemical reactions, including radical-initiated rearrangements, methylation reactions, and reductive cleavage of chemical bonds (34, 35).

**FIG 1:**
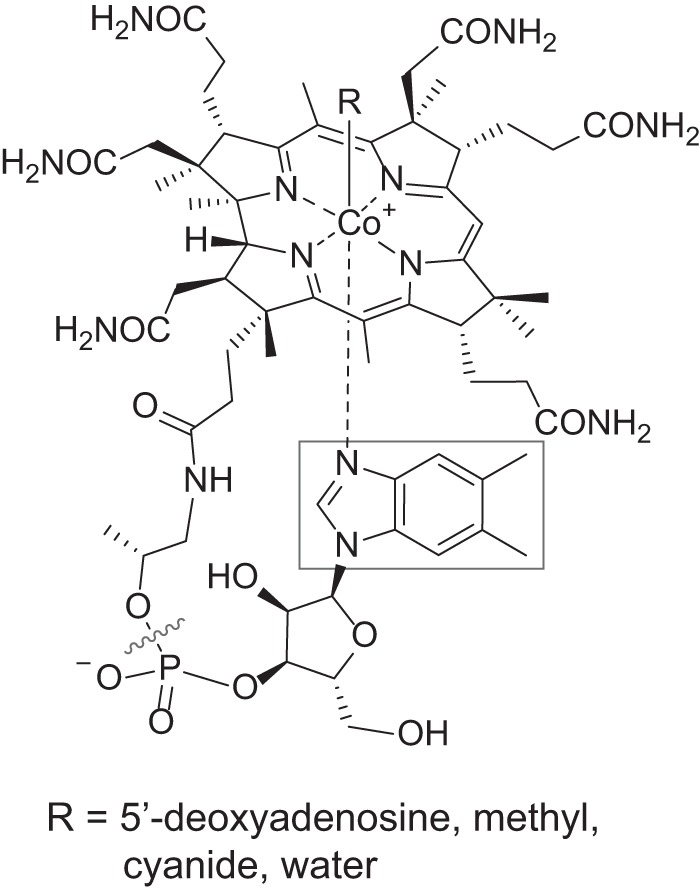
The structure of cobalamin. The lower ligand, boxed, varies in other cobamides. Cobinamide, a cobamide precursor, lacks a nucleotide base (delineated by the wavy line).

**FIG 2:**
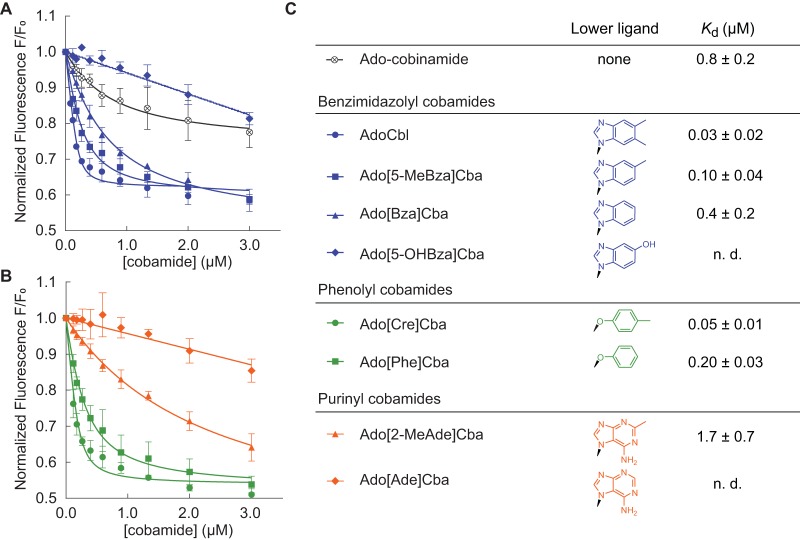
Binding of diverse cobamides to *Sm*MCM (see also Fig. S1). (A and B) Fluorescence decrease of *Sm*MCM when reconstituted with benzimidazolyl cobamides (blue), cobinamide (black), phenolyl cobamides (green), and purinyl cobamides (orange). Data points represent the mean and standard deviation for three technical replicates from a single experiment. (C) *K_d_* values for different cobamides, reported as the average and standard deviation for three or more independent experiments, each consisting of technical triplicates. “n. d.,” not determined, indicating that binding was too weak to determine *K_d_*.

All cobamides share the same core structure (Fig. 1): a corrin ring that coordinates a cobalt ion, a variable “upper” axial ligand (*R* in Fig. 1), and a pseudonucleotide that is covalently attached to the corrin ring through an aminopropanol linker (36) or an ethanolamine linker, in the case of norcobamides (37, 38). The major differences among cobamides are in the structure of the nucleotide base, more commonly referred to as the lower axial ligand for its ability to coordinate the central cobalt ion. In cobalamin, the lower ligand is 5,6-dimethylbenzimidazole (Fig. 1, boxed); in other naturally occurring cobamides, different benzimidazoles, phenolics, and purines constitute the lower ligand (Table 1 and Fig. 2C) (39–43). Phenolyl cobamides are distinct in that they lack the coordinate bond between the lower ligand and cobalt ion.

**TABLE 1:** Abbreviations used for cobamides and upper axial ligands

Abbreviation or prefix	Cobamide name or upper ligand
Abbreviation	
Cbl	Cobalamin
[5-MeBza]Cba	5-Methylbenzimidazolylcobamide
[Bza]Cba	Benzimidazolylcobamide
[5-OHBza]Cba	5-Hydroxybenzimidazolylcobamide
[Cre]Cba	*para*-Cresolylcobamide
[Phe]Cba	Phenolylcobamide
[Ade]Cba	Adeninylcobamide
[2-MeAde]Cba	2-Methyladeninylcobamide
[Pur]Cba	Purinylcobamide
[7-MeBza]Cba	7-Methylbenzimidazolylcobamide
[7-AmBza]Cba	7-Aminobenzimidazolylcobamide
[6-AzaBza]Cba	6-Azabenzimidazolylcobamide
[3-DeazaAde]Cba	3-Deazaadeninylcobamide
[6-MePur]Cba	6-Methylpurinylcobamide
Prefix	
Ado	5′-Deoxyadenosine
CN	Cyanide

10.1128/mBio.01303-19.1 FIG S1 Filtration-based MCM binding assay. UV-Vis spectra of filtrate after preincubation of 10 μM AdoCbl (A), Ado[5-OHBza]Cba (B), and Ado[Ade]Cba (C) with and without *Sm*MCM (15 μM). Download FIG S1, EPS file, 2.3 MB. Copyright © 2019 Sokolovskaya et al. 2019 Sokolovskaya et al. This content is distributed under the terms of the Creative Commons Attribution 4.0 International license.

While cobamides containing different lower ligands share the same chemically reactive moieties, specifically the cobalt center and methyl or 5′-deoxyadenosyl upper axial ligands, they are nonetheless functionally distinct. Culture-based studies have shown that only a subset of cobamides supports a given bacterial metabolism, and uptake or production of other cobamides can inhibit growth (39, 44–49). The requirements of bacteria for particular cobamides are notable given the diversity of cobamides present in host-associated and environmental samples (40–42), coupled with the absence of *de novo* cobamide biosynthesis in more than half of bacteria (33). Despite the biological relevance of cobamide structure, and the prevalence of cobamide use among bacteria (33, 50–52), little is understood about the biochemical mechanisms by which cobamides differentially impact microbial physiology.

The effect of lower ligand structure on the biochemistry of cobamide-dependent enzymes has been studied to a limited extent. In “base-on” enzymes, the lower ligand base coordinates the central cobalt ion of the cobamide, as drawn in Fig. 1 (53–55). Because the lower ligand is part of the catalytic center of the enzyme, lower ligand structure can influence catalysis through a variety of mechanisms (56–58), and cobamides unable to form an intramolecular coordinate bond are catalytically inactive in base-on enzymes (59, 60). In contrast, in “base-off” enzymes the lower ligand is bound by the enzyme more than 10 Å away from the active site (20, 61–70). In a subset of base-off enzymes, referred to as “base-off/His-on,” a histidine residue from the protein coordinates the cobalt ion in place of the lower ligand (61, 63). Despite its distance from the reactive center, lower ligand structure affects the activity of base-off enzymes, as evidenced by the cobamide cofactor selectivity of methionine synthase (71), methylmalonyl coenzyme A (CoA) mutase (MCM) (60, 72), reductive dehalogenases (49), and other enzymes (59, 72, 73). However, the mechanisms by which lower ligand structure affects the biochemistry of base-off cobamide-dependent enzymes remain unclear.

As MCM is one of the most abundant cobamide-dependent enzymes in bacterial genomes (33), and one of the two cobamide-dependent enzymes in humans, we have chosen to study the cobamide selectivity of MCM as a model for base-off/His-on enzymes, all of which share a structurally conserved B_12_-binding domain (63, 74). MCM catalyzes the interconversion of (*R*)-methylmalonyl-CoA and succinyl-CoA, a bidirectional reaction used in propionate metabolism (12, 75, 76), catabolism of branched amino acids and odd-chain fatty acids (76, 77), polyhydroxybutyrate degradation (78), secondary metabolite biosynthesis (79), and autotrophic carbon dioxide fixation (4, 80). MCM-dependent pathways have been harnessed industrially for the bioproduction of propionate, bioplastics, biofuels, and antibiotics (81–88).

The presence of a cobamide lower ligand is required for MCM activity, as evidenced by the observation that adenosylcobinamide, a cobamide intermediate lacking a lower ligand (Fig. 1), does not support MCM activity *in vitro* (89). Three studies provide evidence that MCM is selective for cobamides with particular lower ligands. First, MCM from Propionibacterium shermanii was found to have different apparent *K_m_* values for cobamides, increasing from AdoCbl to Ado[Bza]Cba to Ado[Ade]Cba (refer to Table 1 for full names of cobamides), and MCM from sheep had a higher apparent *K_m_* for Ado[Bza]Cba than AdoCbl (72). Second, *P. shermanii* MCM had a lower apparent *K_m_* for Ado[Cre]Cba than AdoCbl (60). Third, in Sinorhizobium meliloti bacteroids, MCM activity was highest with AdoCbl, intermediate with Ado[Bza]Cba, and absent with Ado[Ade]Cba (90). Each of these studies includes only one or two cobamides other than cobalamin, and understandably so; cobamides are difficult to obtain in high quantities and must be purified from large volumes of bacterial cultures. Because of this, the response of MCM orthologs to the full diversity of cobamides has not been explored, and the mechanistic basis of cobamide selectivity remains unclear.

To investigate the mechanisms by which diverse lower ligands affect MCM function, we conducted *in vitro* binding and activity assays with MCM from S. meliloti (*Sm*MCM). We discovered major differences in the binding affinities of eight naturally occurring cobamides for *Sm*MCM, while cobamide structure affected enzyme activity to a lesser extent. Using six additional cobamides, five of which are novel analogs that have not been observed in nature or described previously, we identified structural elements of lower ligands that are determinants of binding to *Sm*MCM. To probe the hypothesis that enzyme selectivity influences bacterial growth, we characterized the cobamide dependence of S. meliloti growth *in vivo*. By bridging the results of *in vitro* biochemistry of three bacterial MCM orthologs and the cobamide-dependent growth phenotypes of S. meliloti, we have elucidated molecular factors that contribute to the cobamide-dependent physiology of bacteria.

## RESULTS

### Lower ligand structure influences cobamide binding to MCM.

We chose *Sm*MCM as a model to examine how lower ligand structure influences MCM function based on previous work demonstrating its activity as a homodimer encoded by a single gene (91, 92). We purified eight naturally occurring cobamides for *in vitro* studies of this protein and chemically adenosylated each cobamide to produce the biologically active form used by MCM for catalysis. Previous studies showed that binding of cobamides to *P. shermanii* MCM can be detected *in vitro* by measuring quenching of intrinsic protein fluorescence (89). We found that the fluorescence of purified, His-tagged *Sm*MCM also decreased in a dose-dependent manner when the protein was reconstituted with increasing concentrations of AdoCbl (Fig. 2A). The equilibrium dissociation constant (*K_d_*) derived from these measurements, 0.03 ± 0.02 μM (Fig. 2C), is 6-fold lower than the *K_d_* reported for *P. shermanii* MCM (89). Adocobinamide also bound *Sm*MCM, as was observed with *P. shermanii* MCM (89), albeit with over 10-fold-reduced affinity compared to cobalamin (Fig. 2A and C).

We next measured binding of other benzimidazolyl cobamides to *Sm*MCM and found that Ado[5-MeBza]Cba and Ado[Bza]Cba, the cobamides most structurally similar to AdoCbl, also bound the enzyme. However, the absence of one or two methyl groups, respectively, in the lower ligands of these cobamides caused a decrease in binding affinity relative to AdoCbl (Fig. 2A and C). Strikingly, no binding of Ado[5-OHBza]Cba to *Sm*MCM was detected at low-micromolar concentrations. To rule out the possibility that Ado[5-OHBza]Cba binds *Sm*MCM but does not cause a fluorescence quench, we used an alternative, filtration-based, binding assay and observed little to no binding of Ado[5-OHBza]Cba to *Sm*MCM at micromolar concentrations (see Fig. S1A and B in the supplemental material).

We expanded our analysis of *Sm*MCM-cobamide binding selectivity to include cobamides from other structural classes. Both of the phenolyl cobamides tested, Ado[Cre]Cba and Ado[Phe]Cba, bound *Sm*MCM with affinities similar to those of cobalamin and other benzimidazolyl cobamides (Fig. 2B and C). In contrast, the purinyl cobamides Ado[2-MeAde]Cba and Ado[Ade]Cba had lower affinities for *Sm*MCM compared to most benzimidazolyl cobamides (Fig. 2B and C): Ado[2-MeAde]Cba bound *Sm*MCM with ∼20-fold lower affinity than cobalamin, and Ado[Ade]Cba did not bind to any significant extent at micromolar concentrations (verified by the filtration assay [Fig. S1C]). Interestingly, for all three classes of lower ligands, the presence of a methyl substituent promoted binding relative to other cobamides of the same structural class.

### Bacterial MCM orthologs have distinct selectivity.

**FIG 3:**
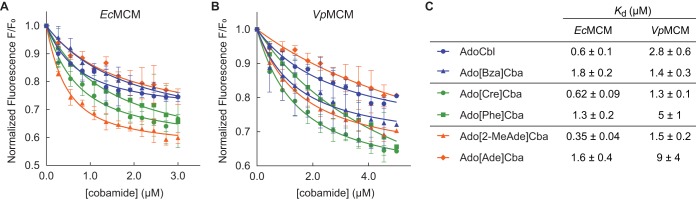
Binding selectivity of diverse MCM orthologs (see also Fig. S2). Fluorescence binding assays with E. coli MCM (A) and *V. parvula* MCM (B). Data points and error bars represent the mean and standard deviation, respectively, for technical triplicates from a single experiment; each replicate consisted of an independent cobamide dilution. *K_d_* values from the fitted curves in panels A and B are reported in panel C; error values reflect the standard error of the curve fit. *K_d_* values for *Vp*MCM binding to Ado[Cre]Cba and AdoCbl and for *Ec*MCM binding to all cobamides were reproduced in independent experiments.

To test whether cofactor-binding selectivity is a general phenomenon across bacterial MCM orthologs, we compared the cobamide-binding profile of *Sm*MCM to that of MCM orthologs from Escherichia coli (*Ec*MCM) and Veillonella parvula (*Vp*MCM). Activity of *Ec*MCM with AdoCbl has been reported both *in vivo* and *in vitro*, although its physiological role in E. coli remains unclear (82, 93). Annotations for two MCM homologs are present in the genome of *V. parvula*, and we purified the one that exhibits MCM activity when expressed in S. meliloti (see Materials and Methods). Because S. meliloti produces cobalamin (94), E. coli produces [2-MeAde]Cba when provided with cobinamide (95), and *V. parvula* produces [Cre]Cba (96), we expected that each ortholog should have distinct cobamide selectivity. Indeed, *Ec*MCM had highest affinity for its native cobamide, Ado[2-MeAde]Cba (Fig. 3A and C). All other cobamides bound with 2- to 3-fold reduced affinities relative to Ado[2-MeAde]Cba. Similarly, *Vp*MCM had a higher affinity for Ado[Cre]Cba, its native cobamide, than AdoCbl (Fig. 3B and C). *Vp*MCM also bound Ado[2-MeAde]Cba and Ado[Bza]Cba with similar affinities. We observed differences between the total changes in fluorescence among cobamides with similar *K_d_* values. This is not unexpected, as protein fluorescence is highly sensitive to local environment and may be affected by subtle conformational differences.

10.1128/mBio.01303-19.2 FIG S2 Sequence comparison of the B_12_-binding domains of MCM orthologs. (A) Sequence alignment of the B_12_-binding domains of MCM orthologs, generated using the MUSCLE alignment tool from EMBL-EBI. Black and gray shading indicates amino acid identity and similarity, respectively. Sequences are colored by “cobamide class” based on cobamides biosynthesized by the organism or predicted cobamide use (C. Belzer, L. W. Chia, S. Aalvink, B. Chamlagain, et al., mBio 8:e00770-17, 2017, https://doi.org/10.1128/mBio.00770-17; Y. Han, A. S. Hawkins, M. W. Adams, and R. M. Kelly, Appl Environ Microbiol 78:6194–6202, 2012, https://doi.org/10.1128/AEM.01312-12; K. C. Mok and M. E. Taga, J Bacteriol 195:1902–1911, 2013, https://doi.org/10.1128/JB.01282-12; T. S. Crofts, E. C. Seth, A. B. Hazra, and M. E. Taga, Chem Biol 20:1265–1274, 2013, https://doi.org/10.1016/j.chembiol.2013.08.006; A. B. Hazra, A. W. Han, A. P. Mehta, K. C. Mok, et al., Proc Natl Acad Sci U S A 112:10792–10797, 2015, https://doi.org/10.1073/pnas.1509132112). Residues numbered above the sequence alignment correspond to residues indicated in Fig. S6. Locus tags of aligned proteins: Homo sapiens AAA59569, Sinorhizobium meliloti AAD13665, Metallosphaera sedula ABP96195, Escherichia coli WP_101348647, Akkermansia muciniphila WP_031931429, Veillonella parvula WP_004694550, Sporomusa ovata WP_021167215. (B) Percent identity matrix of the B_12_-binding domains aligned in panel A, as well as the structural composition of each MCM ortholog: α_2_, homodimer; αβ, heterodimer; α_2_β_2_, heterotetramer. Download FIG S2, EPS file, 1.7 MB. Copyright © 2019 Sokolovskaya et al. 2019 Sokolovskaya et al. This content is distributed under the terms of the Creative Commons Attribution 4.0 International license.

We constructed a sequence alignment of MCM orthologs from diverse organisms known to produce or use various cobamides, in search of amino acid residues that could account for differences in cobamide binding (Fig. S2A). The B_12_-binding domains of diverse MCM orthologs had high overall amino acid identity (38 to 70%). Cases of low identity correlated with differences in the structural configuration of MCM, which occurs in different organisms as a homodimer (92, 93, 97, 98), heterodimer (61, 99–101), or heterotetramer (80, 102) (Fig. S2B). We focused our analysis on residues immediately surrounding the lower ligand in the available crystal structure of Homo sapiens MCM (97) (*Hs*MCM) (Fig. S2A, triangles). For the most part, these residues are highly conserved between orthologs. Interestingly, however, *Hs*MCM residues Phe638, Phe722, and Ala731, which are conserved in *Sm*MCM, are replaced with the more polar residues Tyr, Tyr, and Ser, respectively, in *Ec*MCM (Fig. S2A), which has a higher affinity for purinyl cobamides. Introducing mutations in *Sm*MCM and *Ec*MCM to test the importance of these residues proved challenging, as it resulted in reduced protein solubility and overall impaired cobamide binding (data not shown). Whether or not these residues covary with cobamide selectivity across other MCM orthologs is difficult to interpret because the cobamide selectivity of MCM from most organisms is unknown.

### The lower ligand of cobamides modulates MCM reaction kinetics.

**FIG 4:**
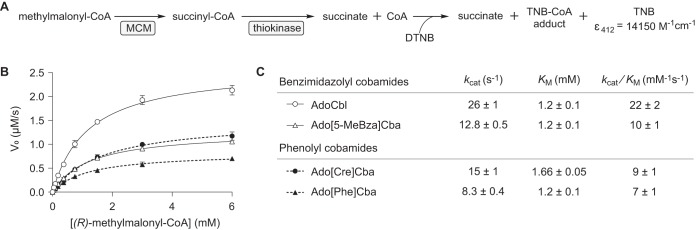
Activity of Sinorhizobium meliloti MCM with different cobamide cofactors. (A) Succinyl-CoA formation was detected using a coupled spectrophotometric assay (138). DTNB, dithionitrobenzoate (Ellman’s reagent); TNB, thionitrobenzoate; CoA, coenzyme A. (B) Michaelis-Menten kinetic analysis of *Sm*MCM reconstituted with various cobamides. Data points and error bars represent the mean and standard deviation, respectively, for three technical replicates from one experiment; each replicate consisted of an independent substrate dilution. (C) Kinetic constants.

We reconstituted *Sm*MCM with saturating amounts of each of the four cobamides that bound with highest affinity and measured conversion of (*R*)-methylmalonyl-CoA to succinyl-CoA under steady-state conditions. Interestingly, the substrate *K_m_* was nearly invariable among the cobamides tested (Fig. 4). Turnover was highest with AdoCbl (26 ± 1 s^−1^) and 2- to 3-fold lower with other cobamides. Thus, all of the cobamides tested supported *Sm*MCM catalysis with modest differences in *k*_cat_. This finding is consistent with a previous observation that adenosylcobinamide-GDP, a cobamide precursor with an extended nucleotide loop and a guanine base, supported activity of *P. shermanii* MCM with only slight catalytic impairment compared to AdoCbl (103).

### MCM-dependent growth of S. meliloti correlates with the binding selectivity of *Sm*MCM for benzimidazolyl and purinyl cobamides, but not phenolyl cobamides.

To assess whether the cobamide-dependent growth of S. meliloti reflects MCM selectivity as observed *in vitro*, we cultured S. meliloti under conditions that require MCM activity. Examination of metabolic pathways encoded in the S. meliloti genome using the KEGG database (104) suggests that the degradation of branched amino acids isoleucine and valine to succinyl-CoA, an intermediate of the citric acid cycle, requires MCM. Indeed, growth of S. meliloti on l-isoleucine and l-valine as the only carbon sources was dependent on the presence of the *bhbA* gene, which encodes MCM (91) (Fig. S3).

10.1128/mBio.01303-19.3 FIG S3 MCM-dependent growth of S. meliloti. Final density (OD_600_), after 72 h of growth in M9 minimal medium, with 4 g/liter l-isoleucine and 4 g/liter l-valine (Ile/Val) or 2 g/liter sucrose. *Sm*MCM is the gene product of the *bhbA* gene. p*bhbA*^+^, complementation of the *bhbA*::Tn*5* mutation with the S. meliloti *bhbA* gene expressed in the pTH1227 vector (J. Cheng, C. D. Sibley, R. Zaheer, and T. M. Finan, 2007, Microbiology 153:375–387, 2007, https://doi.org/10.1099/mic.0.2006/001362-0). The plot shows the mean and standard deviation for three biological replicates from a single experiment. Download FIG S3, EPS file, 1.0 MB. Copyright © 2019 Sokolovskaya et al. 2019 Sokolovskaya et al. This content is distributed under the terms of the Creative Commons Attribution 4.0 International license.

**FIG 5:**
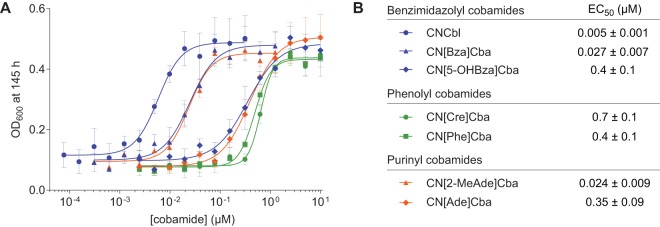
MCM-dependent growth of S. meliloti *cobD*::*gus* Gm^r^ *metH*::Tn*5* Δ*nrdJ* pMS03-*nrdAB_Ec_*^+^ with various cobamides (see also Fig. S3, S4, and S5). (A) Dose dependence of growth based on OD_600_ at 145 h. Data points and error bars represent the mean and standard deviation, respectively, for three biological replicates from a single experiment. (B) EC_50_ values are the average and standard deviation for five or more biological replicates across two or more independent experiments.

We constructed an S. meliloti strain incapable of synthesizing cobalamin and lacking cobamide-dependent enzymes other than MCM to ensure that differential growth could be attributed solely to MCM selectivity for added cobamides (see Materials and Methods). We cultivated this strain with l-isoleucine and l-valine as sole carbon sources in medium supplemented with different cobamides in their cyanylated (CN) form, which is the form typically used for *in vivo* growth assays. Under these growth conditions, the maximum growth yield (optical density at 600 nm [OD_600_]) achieved at high concentrations of all of the cobamides was indistinguishable (Fig. S4A to G). However, the concentration of cobamides required to achieve half of the maximal OD_600_ (50% effective concentration [EC_50_]) differed based on the cobamide provided (Fig. 5). Consistent with the binding data, CNCbl had the lowest EC_50_ value. EC_50_ values for CN[Bza]Cba and CN[2-MeAde]Cba were 5-fold higher than CNCbl, and other cobamides had EC_50_ values 2 orders of magnitude higher than CNCbl.

10.1128/mBio.01303-19.4 FIG S4 MCM-dependent growth of S. meliloti *cobD*::*gus* Gm^r^ *metH*::Tn*5* Δ*nrdJ* pMS03-*nrdAB_Ec_*^+^ with different cobamides. Concentration decreases, by 2-fold dilutions, are shown from pink to blue. Plots show the mean and standard deviation for three biological replicates. Maximum concentrations tested (pink curves) are as follows: CNCbl, 312.5 nM (A); CN[Bza]Cba, 1.25 μM (B); CN[5-OHBza]Cba, 10 μM (C); CN[Ade]Cba, 10 μM (D); CN[2-MeAde]Cba, 1.25 μM (E); CN[Phe]Cba, 10 μM (F); CN[Cre]Cba, 10 μM (G); and CN[Pur]Cba, 10 μM (H). Download FIG S4, TIF file, 2.2 MB. Copyright © 2019 Sokolovskaya et al. 2019 Sokolovskaya et al. This content is distributed under the terms of the Creative Commons Attribution 4.0 International license.

10.1128/mBio.01303-19.5 FIG S5 Quantification of cobamides internalized by S. meliloti *cobD*::*gus* Gm^r^ *metH*::Tn*5* Δ*nrdJ* pMS03-*nrdAB_Ec_*^+^. (A) Cellular cobamide concentrations following 48 h of growth in M9 sucrose with the indicated concentrations of cobamides added to the medium. The range of concentrations measured in cell pellets was determined by HPLC analysis of corrinoid extractions from two or more independent experiments, each including biological duplicates. (B) A graphic illustrating the concentrations of different cobamides at which intracellular cobamide concentrations are comparable. Internalization data (colored) are overlaid onto dose-response curves from Fig. 5 (black). Five points indicate cobamide concentrations at which cobamides were extracted and quantified; dotted lines connect conditions under which intracellular concentrations of different cobamides are approximately equal. Download FIG S5, EPS file, 1.9 MB. Copyright © 2019 Sokolovskaya et al. 2019 Sokolovskaya et al. This content is distributed under the terms of the Creative Commons Attribution 4.0 International license.

With the notable exception of the phenolyl cobamides, differences in the EC_50_ values of cobamides *in vivo* qualitatively correlated with the binding selectivity that we observed *in vitro* (Fig. 2). Among benzimidazolyl cobamides, EC_50_ values increased from cobalamin to [Bza]Cba to [5-OHBza]Cba, consistent with the relative binding affinities of these cobamides. Similarly, [2-MeAde]Cba, which had an intermediate binding affinity for *Sm*MCM, had a lower EC_50_ value than [Ade]Cba, which did not bind to *Sm*MCM at low-micromolar concentrations *in vitro.* The ability of [5-OHBza]Cba and [Ade]Cba to support growth suggests that these cobamides can bind *Sm*MCM at concentrations higher than those tested *in vitro*; a control experiment with an S. meliloti strain lacking MCM rules out the possibility that high concentrations of cobamides (10 μM) abiotically enable growth on isoleucine and valine (Fig. S3).

We considered the possibility that differences in cobamide internalization by S. meliloti could also influence the EC_50_ measurements shown in Fig. 5. When S. meliloti cultures were supplemented with equimolar amounts of CNCbl, CN[Ade]Cba, or CN[Cre]Cba, the concentration of cobalamin extracted from the cellular fraction was 2- to 3-fold higher than [Ade]Cba and 5- to 6-fold higher than [Cre]Cba (Fig. S5A). This result suggests that cobamides are differentially internalized or retained by the cells. However, MCM-dependent growth does not correlate with intracellular cobamide concentrations, as intracellular concentrations of cobalamin comparable to those of [Ade]Cba and [Cre]Cba supported S. meliloti growth to high densities (Fig. S5). Therefore, the high EC_50_ of CN[Ade]Cba relative to CNCbl is more likely attributable to enzyme selectivity. Additional factors that could explain the high EC_50_ values of the phenolyl cobamides are considered in Discussion.

### Identification of structural elements that interfere with cobamide binding.

Given the apparent importance of MCM cobamide binding selectivity for the cobamide-dependent growth of S. meliloti, we pursued a more mechanistic understanding of how lower ligand structure affects cobamide binding. When cobamides are bound to MCM, the lower ligand is surrounded by protein residues (61, 97). Therefore, the reduced affinity of certain cobamides for the enzyme could be a result of exclusion of their lower ligands from this binding pocket because of steric or electrostatic repulsion. We hypothesized that the poor binding of the purinyl cobamides Ado[Ade]Cba and Ado[2-MeAde]Cba is due to the presence of the exocyclic amine based on several observations: (i) Ado[5-OHBza]Cba, which also contains a polar functional group, had impaired binding to *Sm*MCM (Fig. 2A and C). (ii) In the crystal structure of *Hs*MCM (97), residues Phe722 and Ala731, which are conserved in *Sm*MCM, would be expected to electrostatically occlude the exocyclic amine of [Ade]Cba (Fig. S6A, asterisk). Based on sequence alignment (Fig. S2A), polar residues Tyr and Ser would be expected to occupy the corresponding positions in *Ec*MCM, which has higher affinity for purinyl cobamides. (iii) Structural modeling of Ado[Ade]Cba bound to *Hs*MCM, which shares 59% amino acid identity to *Sm*MCM in the B_12_-binding domain, suggests significant displacement of the adenine lower ligand relative to the lower ligand of AdoCbl, in the direction that would be consistent with steric or electrostatic repulsion of the exocyclic amine by surrounding residues (Fig. S6B to D).

10.1128/mBio.01303-19.6 FIG S6 The lower ligand binding pocket of MCM. (A) Residues surrounding the lower ligand of AdoCbl in the X-ray crystal structure of Homo sapiens MCM (*Hs*MCM) (PDB 2XIQ, gray). A model of *Sm*MCM, generated by sequence alignment and threading using Swiss-Prot, is overlaid in white. The asterisk marks the expected position of the exocyclic amine of [Ade]Cba. (B) Surface depiction of the lower ligand binding pocket of *Hs*MCM bound to cobalamin, after performing a constrained energy minimization. As expected, no major differences were observed between the energy-minimized model and the original structure. (C) Surface depiction of the lower ligand binding pocket of *Hs*MCM modeled with [Ade]Cba bound, generated by changing the structure of the lower ligand in panel B and performing a constrained energy minimization. (D) Overlay of the structural models in panels B and C. Download FIG S6, TIF file, 2.0 MB. Copyright © 2019 Sokolovskaya et al. 2019 Sokolovskaya et al. This content is distributed under the terms of the Creative Commons Attribution 4.0 International license.

**FIG 6:**
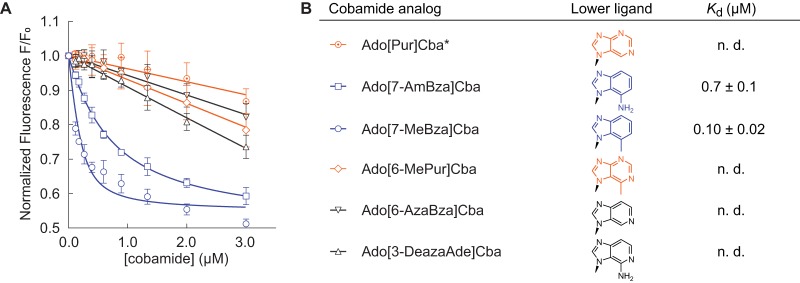
Binding of cobamide analogs to *Sm*MCM (see also Fig. S8). (A) Fluorescence decrease of *Sm*MCM when reconstituted with benzimidazolyl (blue), purinyl (orange), and azabenzimidazolyl (black) cobamide analogs. Data points represent the mean and standard deviation for three technical replicates from a single experiment. (B) *K_d_* values for different cobamides, reported as the average and standard deviation for three or more independent experiments, each consisting of technical triplicates. “n. d.,” not determined, indicating that binding was too weak to determine *K_d_*. The asterisk indicates that, while it was unreported at the time of our study, [Pur]Cba was discovered to be the cobamide naturally produced by Desulfitobacterium hafniense (39).

To test the importance of the exocyclic amine of adenine in cofactor exclusion, we produced an unsubstituted purinyl cobamide, Ado[Pur]Cba (39). Ado[Pur]Cba also had low affinity for *Sm*MCM (Fig. 6A and B), suggesting that the exocyclic amine of adenine is not a major cause of binding exclusion. Consistent with this result, a novel benzimidazolyl cobamide, Ado[7-AmBza]Cba, bound *Sm*MCM with comparable affinity as Ado[Bza]Cba, despite being functionalized with an exocyclic amine (Fig. 6A and B). Rather, these results suggest that the presence of nitrogens in the six-membered ring of the lower ligand interferes with binding. To test this hypothesis directly, we produced three cobamide analogs with at least one nitrogen in the six-membered ring of the lower ligand base. Comparison of the binding of Ado[7-MeBza]Cba and Ado[6-MePur]Cba (Fig. 6A and B) supported a role of ring nitrogens in binding inhibition, and comparison of binding affinities between Ado[Bza]Cba and Ado[6-AzaBza]Cba (105) (Fig. 2A and C and Fig. 6A and B, respectively), and between Ado[7-AmBza]Cba and Ado[3-DeazaAde]Cba (Fig. 6A and B), revealed that a single nitrogen atom in the six-membered ring of the lower ligand was sufficient to severely impair binding.

10.1128/mBio.01303-19.8 FIG S8 Spectral analysis of novel cobamide analogs. (Left) Absorbance spectra of cyanylated cobamides, recorded during HPLC analysis of crude extracts. Labels indicate local absorbance maxima. (Right) Mass spectra from HPLC-MS analysis of cobamides following adenosylation. Cobamides containing impurities were further purified by HPLC. Each lower ligand is drawn in the same orientation as adenine in [Ade]Cba (B. Hoffmann, M. Oberhuber, E. Stupperich, H. Bothe, et al., J Bacteriol 182:4773–4782, 2000, https://doi.org/10.1128/jb.182.17.4773-4782.2000), though it is formally possible that the lower ligands are attached in the opposite orientation (e.g., at N3 on [7-MeBza]Cba). Download FIG S8, EPS file, 2.0 MB. Copyright © 2019 Sokolovskaya et al. 2019 Sokolovskaya et al. This content is distributed under the terms of the Creative Commons Attribution 4.0 International license.

As it was recently discovered to be a naturally occurring cobamide (39), we tested the MCM-dependent growth of S. meliloti with [Pur]Cba. [Pur]Cba had a high EC_50_ value of 0.6 ± 0.2 μM (Fig. S4H), further supporting the correlation between binding and growth that we previously observed for benzimidazolyl and purinyl cobamides.

## DISCUSSION

Cobamides are distinct from other cofactors in their extensive structural diversity, with over a dozen forms that differ in the lower ligand base and nucleotide loop. How cobamide lower ligand structure influences the activity of cobamide-dependent enzymes has not been extensively explored. Here, we report a systematic analysis of the effects of cobamide lower ligand structure on the function of a model cobamide-dependent enzyme, MCM. Our results show that MCM exhibits varied affinities for different cobamides and that this selectivity is linked to the physiology of the organism.

Our results show that the major determinant of cobamide selectivity in *Sm*MCM is binding, with small changes in the lower ligand capable of dramatically altering the binding affinity of a cobamide. One explanation for these differences is that the chemical compatibility between the lower ligand base and the binding pocket of the protein strongly influences the binding affinity of cobamides; repulsion of the lower ligand on the basis of electrostatics could reduce the binding affinity of cobamides to MCM. While the structure of *Sm*MCM has not been determined, a model generated by sequence alignment to *Hs*MCM suggested a highly hydrophobic lower ligand binding pocket. Consistent with this, we observed higher affinity of cobamides with hydrophobic lower ligands to *Sm*MCM, as well as interference of ring nitrogens with cobamide binding.

On the other hand, sequence alignments suggested that many of the hydrophobic residues predicted to immediately surround the lower ligand are conserved between diverse MCM orthologs that differ in cobamide selectivity. Assuming that the arrangement of the lower ligand binding pocket is similar across MCM orthologs, this suggests that interactions within the lower ligand binding pocket are not sufficient to account for selectivity. In a similar vein, examination of the residues surrounding the lower ligand in the cobamide-bound structures of reductive dehalogenases does not reveal the basis of exclusion of certain cobamides (49). These observations suggest that the lower ligand may have an unknown role in the binding of cobamides to MCM. Consistent with this idea, studies of the kinetics and pH dependence of AdoCbl binding to *P. shermanii* MCM suggest a preassociation step, wherein a cofactor-protein complex is formed prior to displacement of the lower ligand of the cofactor by a histidine residue in the protein (89). The nature of this complex is unknown, but potential interactions between the lower ligand and this conformation of the enzyme could provide an opportunity for lower ligand structure to impact the outcome of binding.

Our analysis of MCM orthologs from E. coli and *V. parvula* demonstrates that variations in cobamide selectivity have evolved in organisms with different physiologies. The cobamide selectivity patterns in the three MCM orthologs we examined correlate with the physiologies of the bacteria in two ways. First, in all three cases, each MCM ortholog has highest affinity for the native cobamide produced by the organism, suggesting that cobamide biosynthesis and selectivity of cobamide-dependent enzymes have coevolved. Second, *Sm*MCM is more selective than *Ec*MCM and *Vp*MCM, which is consistent with differences in cobamide biosynthesis, acquisition, and use in these organisms. S. meliloti synthesizes cobalamin *de novo* and is incapable of attaching purinyl and phenolyl lower ligands to cobamide precursors (96). Thus, its cobamide-dependent enzymes have likely evolved to function best with cobalamin. In contrast, E. coli does not synthesize cobamides *de novo* and instead relies on the importer BtuBFCD to acquire cobamides from the environment (106, 107). Alternatively, E. coli can produce a variety of benzimidazolyl and purinyl cobamides when provided with precursors (95), making the ability to use multiple cobamides likely advantageous. Like S. meliloti, *V. parvula* synthesizes cobamides *de novo* but can produce both benzimidazolyl and phenolyl cobamides (96, 108) and also encodes membrane transport components adjacent to cobalamin riboswitches (109), which are likely to be cobamide importers (52, 110). Thus, the ability of *Vp*MCM to bind diverse cobamides is similarly consistent with its physiology.

Relative to cobamide binding selectivity, our results suggest that effects of lower ligand structure on the catalytic activity of MCM are minor. Among the cobamides we tested, the maximum differences in *Sm*MCM turnover were 3-fold. We did not observe inhibition of MCM activity with any cobamides, in contrast to the strong inhibition that has been observed with analogs containing variations in the upper ligand or central metal, known as antivitamins (111–113).

**FIG 7:**
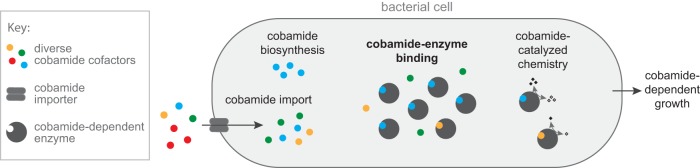
Model describing biochemical determinants of cobamide-dependent growth in bacteria. Cobamides differentially impact bacterial growth due to selective cobamide import and biosynthesis, cobamide-binding selectivity of cobamide-dependent enzymes, and cobamide-dependent catalysis. For MCM-dependent growth of S. meliloti, cobamide-binding selectivity is most strongly correlated with cobamide-dependent growth of the organism.

In addition to elucidating the biochemical basis of cobamide selectivity in MCM, a major aim of our work was to link biochemical selectivity with cobamide-dependent growth. Our results with benzimidazolyl and purinyl cobamides support the hypothesis that enzyme selectivity is a major determinant of cobamide-dependent growth. Interestingly, although phenolyl cobamides bound *Sm*MCM with high affinity and supported catalysis *in vitro*, high concentrations were required to support growth of S. meliloti. This discrepancy can be partially explained by poorer internalization or retention of these cofactors compared to cobalamin (see Fig. S5 in the supplemental material). The observation that the intracellular cobamide concentrations were 50- to 190-fold greater than the amount added to the growth medium (Fig. S5A) suggests that cobamides could be internalized by an uptake mechanism that favors cobalamin, distinct from both BtuBFCD and ECF-CbrT (114, 115), both of which are absent from S. meliloti. Thus, we propose a model in which the cobamide-dependent growth of bacteria is influenced not only by binding selectivity of cobamide-dependent enzymes but also by cobamide import (Fig. 7). The lower effectiveness of phenolyl cobamides in supporting growth of S. meliloti could additionally be explained by inefficient adenosylation of these cobamides *in vivo*, as MCM requires the adenosyl upper axial ligand for activity. Whether or not adenosyltransferase enzymes, specifically CobA and PduO (116, 117) in S. meliloti, are selective with respect to lower ligand structure is unknown.

We and others have proposed the possibility of manipulating microbial communities using cobamides by taking advantage of the differential cobamide-dependent growth of bacteria (39, 118–120). Cobamides are predicted to mediate microbial interactions that are critical to the assembly of complex communities (33, 41, 45, 50, 121–124), so the ability to selectively inhibit or promote the growth of particular species using corrinoids with various lower ligands could be applied to alter the composition of microbial communities in ways that could promote environmental and human health. This possibility hinges on the ability to predict which cobamides support or inhibit growth of an organism of interest, which requires an understanding of the major biochemical determinants of growth. We observed here that the cobamide binding selectivity of a model base-off cobamide-dependent enzyme correlates with growth to a large extent. Thus, uncovering protein residues that confer selectivity would enable prediction of selectivity in cobamide-dependent enzymes, thereby facilitating prediction of the cobamide requirements of organisms of interest. Furthermore, our results suggest that additional steps of cobamide trafficking may be important determinants of cobamide-dependent growth. Future studies to understand how these various steps depend on cobamide structure will ultimately allow us to better understand, predict, and manipulate microbial interactions.

## MATERIALS AND METHODS

### Chemical reagents.

Chemicals were obtained from the sources indicated: 5′-chloro-5′-deoxyadenosine, Santa Cruz Biotechnology; 7-methylbenzimidazole, Accela; 5-methyl-1H-benzimidazole, Acros Organics; phenol, J. T. Baker; zinc metal, Fisher Scientific; 5-methoxybenzimidazole, purine, and *para*-cresol, Alfa Aesar; methylmalonyl-CoA, methylmalonic acid, coenzyme A, adenosylcobalamin (coenzyme B_12_), cyanocobalamin, dicyanocobinamide, 6-methylpurine, 1H-imidazo[4,5-c]pyridine-4-amine (3-deazaadenine), benzimidazole, adenine hemisulfate, 5-azabenzimidazole, 1H-benzo[d]imidazol-7-amine (7-aminobenzimidazole), 2-methyl-1H-purine-6-amine (2-methyladenine), and bovine serum albumin (BSA), Sigma.

### Molecular cloning, protein expression, and purification.

*Sm*MCM (locus SM_b20757, *bhbA*) was expressed from the pET28a vector, with an N-terminal hexahistidine (6×His) tag, in E. coli BL21(DE3)pLysS (cloning primers are listed in Table S1 in the supplemental material). The expression strain was grown to an optical density at 600 nm (OD_600_) of 0.6 to 0.8 at 37°C, cooled on ice for 15 min, and induced with 1 mM isopropyl-β-d-thiogalactopyranoside (IPTG) for 2.5 h at 37°C. Cells were lysed by sonication in 25 mM Tris-HCl, pH 8.0, 300 mM NaCl, 10 mM imidazole, with 0.5 mM phenylmethylsulfonyl fluoride (PMSF), 1 μg/ml leupeptin, 1 μg/ml pepstatin, and 1 mg/ml lysozyme. Clarified lysate was treated with 0.05% polyethyleneimine. An Äkta Pure 25 fast protein liquid chromatography (FPLC) system was used to purify the protein over a GE 5-ml HisTrap HF column, using a gradient of 21 to 230 mM imidazole in the lysis buffer. Purified protein was dialyzed into 25 mM Tris-HCl, pH 8.0, 300 mM NaCl, 10% glycerol and concentrated with a Vivaspin 10,000-molecular-weight-cutoff (MWCO) protein concentrator. Purity was analyzed by SDS-PAGE (Fig. S7), and protein concentration was determined by *A*_280_ using the theoretical extinction coefficient 55,810 M^−1^ cm^−1^ (125). *Ec*MCM (locus b2917, *scpA*, previously *sbmA*) was expressed with an N-terminal 6×His tag from a pET28a vector in E. coli BL21(DE3), by induction at an OD_600_ of 0.6 to 0.8 with 0.1 mM IPTG, for 3.5 h at 30°C. The protein was purified as described above, and the final concentration was determined by Coomassie blue-stained SDS-PAGE (Fig. S7), using BSA as a standard.

10.1128/mBio.01303-19.7 FIG S7 SDS-PAGE analysis of purified MCM orthologs. Gels are Coomassie blue stained. Download FIG S7, TIF file, 2.0 MB. Copyright © 2019 Sokolovskaya et al. 2019 Sokolovskaya et al. This content is distributed under the terms of the Creative Commons Attribution 4.0 International license.

10.1128/mBio.01303-19.9 TABLE S1 Cloning primers. Vector-derived sequences are in lowercase letters. Download Table S1, DOCX file, 0.01 MB. Copyright © 2019 Sokolovskaya et al. 2019 Sokolovskaya et al. This content is distributed under the terms of the Creative Commons Attribution 4.0 International license.

The *V. parvula* genome has two MCM annotations: a heterotetramer (loci Vpar_RS06295 and Vpar_RS06290) and a heterodimer (loci Vpar_RS09005 and Vpar_RS09000). The functionality of both homologs was tested by complementation in S. meliloti. The two putative *Vp*MCM enzymes were cloned into the pTH1227 vector and transferred by conjugation into an S. meliloti *bhbA*::Tn*5* mutant. Complementation was assessed by growth in M9 liquid medium containing l-isoleucine and l-valine (see “S. meliloti growth assays” below for additional details). S. meliloti coexpressing Vpar_RS09005 and Vpar_RS09000 showed identical growth to a strain expressing *Sm*MCM from pTH1227 and was selected for *in vitro* studies.

The α subunit of *Vp*MCM (encoded by Vpar_RS09005) was expressed with an N-terminal 6×His tag from the pET-Duet expression vector in E. coli BL21(DE3). Protein expression was induced with 520 μM IPTG for 6 h at 30°C. The protein was batch purified by nickel affinity and subsequently purified by FPLC using a HiTrapQ column with an NaCl gradient from 50 to 500 mM in 20 Tris-HCl, pH 8.0, 10% glycerol. The β subunit of *Vp*MCM (encoded by Vpar_RS09000) was expressed separately with an N-terminal 6×His tag from the pET-Duet expression vector in E. coli BL21(DE3). Expression was induced with 1 mM IPTG for 22 h at 16°C, and the protein was purified using nickel-affinity chromatography as described for *Sm*MCM. Purified protein was dialyzed into 25 mM Tris-HCl, pH 8.0, 300 mM NaCl, 10% glycerol, and 1 mM β-mercaptoethanol. Concentration of pure α and β subunits (Fig. S7) was determined by absorbance at 280 nm (*A*_280_) using the theoretically calculated extinction coefficients 75,290 M^−1^ cm^−1^ and 74,260 M^−1^ cm^−1^, respectively (125). Equimolar amounts of α and β subunits were combined during the setup of fluorescence binding assays.

E. coli thiokinase containing an N-terminal 6×His tag was expressed from a vector provided by Gregory Campanello from the laboratory of Ruma Banerjee. Expression was induced with 1 mM IPTG in E. coli BL21(DE3)pLysS at 28°C for 3 h. The protein was purified as a heterodimer using nickel-affinity chromatography as described above. His-tagged Rhodopseudomonas palustris MatB (126) was expressed from a pET16b expression plasmid provided by Omer Ad from the laboratory of Michelle Chang. The protein was overexpressed in E. coli BL21(DE3) at 16°C overnight, after induction with 1 mM IPTG, and purified by nickel-affinity chromatography as indicated above. Thiokinase and MatB concentrations were determined by Coomassie blue-stained SDS-PAGE, using BSA as a standard.

### Guided biosynthesis, extraction, and purification of cobamides.

Sporomusa ovata strain DSM 2662 was used for the production of its native cobamide, [Cre]Cba, and for production of [Phe]Cba, [5-MeBza]Cba, [Bza]Cba, [5-OHBza]Cba, [7-MeBza]Cba, and [7-AmBza]Cba, by guided biosynthesis as previously described (44). 5-OHBza was synthesized as described previously (96). Salmonella enterica serovar Typhimurium strain LT2 and Propionibacterium acidipropionici strain DSM 20273 were used for production of [Ade]Cba (47, 127). [2-MeAde]Cba, [Pur]Cba, [6-AzaBza]Cba, [3-DeazaAde]Cba, and [6-MePur]Cba were produced by guided biosynthesis in *P. acidipropionici*. Cobamides were extracted as previously described (47) and purified by high-performance liquid chromatography (HPLC) using previously published methods (47, 96, 128) as well as additional methods listed in Table S2. In many cases, more than one method was required to achieve high purity. Identity of cobamides was confirmed by liquid chromatography (LC) coupled to mass spectrometry (MS) using an Agilent 1260 LC/6120 quadrupole MS instrument. The orientation of the lower ligands of [7-MeBza]Cba, [7-AmBza]Cba, [3-DeazaAde]Cba, and [6-MePur]Cba is likely to be analogous to the lower ligand orientation in purinyl cobamides (127, 129). This assumption is supported by the absorbance spectra of these cobamides under acidic conditions, which reveal a base-on conformation (Fig. S8); the opposite orientation of the amino and methyl substituents would create steric interference between the lower ligand and corrin ring, which would be expected to weaken the coordination bond and favor a base-off conformation, especially at low pH. The orientation of the lower ligands in [Pur]Cba and [6-AzaBza]Cba was not determined.

10.1128/mBio.01303-19.10 TABLE S2 HPLC methods. Download Table S2, DOCX file, 0.01 MB. Copyright © 2019 Sokolovskaya et al. 2019 Sokolovskaya et al. This content is distributed under the terms of the Creative Commons Attribution 4.0 International license.

### Chemical adenosylation of cobamides.

Cobamide adenosylation was performed as previously described (128, 130). Briefly, cobamides at concentrations of 0.5 to 1 mM were reduced with activated zinc metal under anaerobic conditions, with vigorous stirring for 0.5 to 2 h. 5′-Chloro-5′-deoxyadenosine was added, and adenosylation was allowed to proceed for 1 to 3 h in the dark. The progress of the reaction was monitored by HPLC. Following adenosylation, cobamides were desalted using a C_18_ SepPak (Waters), purified by HPLC, desalted again, dried, and stored at −20°C or −80°C.

### Cobamide quantification.

Purified cobamides were dissolved in water and quantified by UV-Vis spectrophotometry on a BioTek Synergy 2 plate reader using the following extinction coefficients: for cyanylated benzimidazolyl cobamides, ɛ_518_ = 7.4 × 10^3^ M^−1^ cm^−1^ (131); for cyanylated purinyl cobamides, ɛ_548_ = 7.94 × 10^3^ M^−1^ cm^−1^ (132); for cyanylated phenolyl cobamides, ɛ_495_ = 9.523 × 10^3^ M^−1^ cm^−1^ (133); for adenosylated benzimidazolyl cobamides (AdoCbl, Ado[5-MeBza]Cba, Ado[Bza]Cba, Ado[5-OHBza]Cba, Ado[7-MeBza]Cba, and Ado[7-AmBza]Cba), which are predominantly base-on in water, ɛ_522_ = 8.0 mM^−1^ cm^−1^ (131); for adenosylated purinyl cobamides (Ado[Ade]Cba, Ado[2-MeAde]Cba, and Ado[Pur]Cba), which are predominantly base-off in water, and phenolyl cobamides (Ado[Cre]Cba and Ado[Phe]Cba), which are base-off, ɛ_458_ = 8.8 mM^−1^ cm^−1^ (132); for adenosylated azabenzimidazolyl cobamides (Ado[3-DeazaAde]Cba, Ado[6-AzaBza]Cba, and Ado[6-MePur]Cba), which are a mixture of base-on and base-off in water, the concentration was estimated from the average of concentrations calculated using the extinction coefficients above.

### Fluorescence binding assays.

An *in vitro* assay previously described for measuring binding of AdoCbl to *P. shermanii* MCM (89) was adapted to a 96-well format: MCM (0.2 μM) was combined with a range of cobamide concentrations (as specified in each experiment) in a black 96-well plate in 50 mM potassium phosphate, pH 7.5, with 1 mM dithiothreitol (DTT), on ice. All steps involving cobamides were conducted in the dark. The plate was centrifuged for 1 min at 3,800 rpm to level the surface of the liquid in each well. The plate was then incubated for 40 min at 30°C to allow binding, with a brief shaking step after 30 min. Preliminary experiments showed that this time is sufficient for equilibration. Following incubation, fluorescence emission at 340 nm (5-nm slit width) was measured upon excitation at 282 nm (5-nm slit width) using a Tecan Infinite M1000 Pro plate reader. Fluorescence, normalized to the initial value, was plotted as a function of cobamide concentration, and fitted to the following equation (134):

$$\frac{F}{F_{0}}=1+\frac{\Delta F_{\text{max}}}{F_{0}}\frac{([E]+[L]+K_{d})-\sqrt{{\left(\left[E\right]+\left[L\right]+K_{d}\right)}^{2}-4[E][L]}}{2[E]}$$

where *F* is fluorescence, *F*_0_ is initial fluorescence, [*E*] is total enzyme concentration, [*L*] is total ligand concentration, and *K_d_* is the binding dissociation constant.

### Filtration binding assay.

Cobamides (10 μM) with and without MCM (15 μM) were incubated in 100 mM Tris, 50 mM phosphate, pH 7.5, at 30°C for 40 min, transferred to Nanosep 10K Omega centrifugal devices (Pall Corporation), and centrifuged for 5 min at 13,900 × *g* to separate unbound cobamides from enzyme-bound cobamides. The UV-Vis spectra of the filtrates were recorded on a BioTek Synergy 2 plate reader.

### Structural modeling.

A model of *Sm*MCM was generated using the Swiss-Model software (135) based on the known crystal structure of Homo sapiens MCM (*Hs*MCM) (PDB ID 2XIJ) (97). No major differences were observed in the B_12_-binding domain between *Sm*MCM models generated from *Hs*MCM and Propionibacterium freudenreichii MCM (PDB ID 4REQ) (61).

Maestro (136) was used to generate a model of *Hs*MCM bound to [Ade]Cba. The initial structure of *Hs*MCM bound to cobalamin (PDB ID 2XIQ) (97) was prepared using standard methods. A constrained energy minimization (atoms within 10 Å of cobalamin freely moving; atoms within a second 10-Å shell constrained by a force constant of 200; remaining structure frozen) was performed using MacroModel (137). The structure of the lower ligand was then modified to adenine, and the constrained energy minimization was repeated to generate a model of the lower ligand binding pocket bound to [Ade]Cba.

### Enzymatic synthesis of (*R*)-methylmalonyl-CoA.

(*R*)-Methylmalonyl-CoA synthesis reaction mixtures contained the following in 10 ml: 100 mM sodium phosphate (pH 7.5), 20 mM MgCl_2_, 5 mM ATP, 10 mM methylmalonic acid, 2 mM coenzyme A, 5 mM β-mercaptoethanol, and 1.5 μM purified MatB protein. After combining ingredients on ice, the reaction mixture was incubated at 37°C for 1 h. The reaction mixture was then frozen in liquid nitrogen and lyophilized. To purify (*R*)-methylmalonyl-CoA, the dried reaction mixture was resuspended in 3.2 ml water, the protein was precipitated with 200 μl trichloroacetic acid, precipitate was pelleted, supernatant was neutralized with 200 μl of 10 M NaOH, and salts and remaining starting materials were removed using a C_18_ SepPak column (Waters) (loaded in 0.1% formic acid and washed with water, methylmalonyl-CoA was eluted with 50% methanol in water). Formation of (*R*)-methylmalonyl-CoA was initially verified by ^1^H nuclear magnetic resonance (NMR) and in subsequent preparations by HPLC (Table S2). The concentration of (*R*)-methylmalonyl-CoA was determined using an extinction coefficient of 12.2 mM^−1^ cm^−1^ at 259 nm.

### MCM activity assays.

A thiokinase-coupled, spectrophotometric MCM activity assay was adapted from previous work (138), except that ADP was used instead of GDP, and the experiment was conducted in 96-well plates. Final concentrations of reagents in the assays were as follows: Tris-phosphate buffer (pH 7.5), 100 mM Tris, 50 mM phosphate; dithionitrobenzoate (DTNB), 400 μM; ADP, 1 mM; MgCl_2_, 10 mM; (*R*)-methylmalonyl-CoA, 0 to 4 mM; thiokinase, 5 μM; MCM, 50 nM; and cobamides, 2 μM. Preliminary experiments were conducted to ensure that concentrations of thiokinase, DTNB, and cobamides were not rate limiting.

Three separate mixes were prepared, all in 1× Tris-phosphate buffer: an assay mix containing DTNB, ADP, and MgCl_2_; a substrate mix containing (*R*)-methylmalonyl-CoA; and an enzyme mix containing thiokinase, MCM, and cobamides. All steps involving cobamides were conducted in the dark. The assay and enzyme mixes were prepared as a master mix and aliquoted into 96-well plates; substrate mixes were prepared in individual wells, in triplicate. All components were incubated at 30°C for 40 min to equilibrate temperature and allow prebinding of cobamides and MCM. After incubation, one replicate at a time, the substrate mix was added to the assay mix, followed by the enzyme mix. Absorbance at 412 nm (*A*_412_) was recorded immediately after addition of enzyme and for 1 to 3 min, every 3 s, on a BioTek Synergy 2 plate reader. The increase in *A*_412_ in reaction mixtures lacking substrate was subtracted from all readings, to account for reactivity of DTNB with thiols on protein surfaces. *A*_412_ values were converted to concentration of free CoA using a path-length correction determined for the reaction volume and extinction coefficient of 14,150 M^−1^ cm^−1^.

### S. meliloti growth assays.

MCM-dependent growth experiments were performed with S. meliloti strain Rm1021 *cobD*::*gus* Gm^r^ *metH*::Tn*5* Δ*nrdJ* pMS03-*nrdAB_Ec_*^+^, which lacks cobamide-dependent enzymes other than MCM and does not synthesize cobalamin. *cobD* is required for cobalamin biosynthesis (139), *metH* encodes methionine synthase (139–141), and *nrdJ* encodes ribonucleotide reductase (142). Because *nrdJ* is essential, the E. coli cobamide-independent ribonucleotide reductase encoded by *nrdA* and *nrdB* was expressed from the pMS03 plasmid (143). The strain was precultured in M9 medium (144) (modified concentration of MgSO_4_: 1 mM) containing 0.1% sucrose, 2 g/liter isoleucine, 2 g/liter valine, 1 g/liter methionine, and 20 μg/ml gentamicin, with shaking at 30°C. After 2 days, cells were washed and diluted to an OD_600_ of 0.02 into M9 medium containing 4 g/liter isoleucine, 4 g/liter valine, 1 g/liter methionine, 20 μg/ml gentamicin, and cobamides at various concentrations as indicated for each experiment, in 384-well plates. The plates were incubated at 30°C for 145 h in a BioTek Synergy 2 plate reader with linear shaking at 1,140 cpm. OD_600_ was measured in 1-h increments.

For quantification of intracellular cobamides in S. meliloti, the strain above was precultured as described above, diluted into 50 ml of M9 medium containing 0.2% sucrose and various cobamides, and grown for 48 h (until OD_600_ reached 0.6 to 0.8). Cobamides were extracted from cell pellets as previously described (47), using 5 ml of methanol containing 500 μg of potassium cyanide. A partial purification by means of a wash step with 20% methanol in water was included during the SepPak desalting procedure. Extracted cobamides were quantified by HPLC using peak areas at 525 nm and external standard curves, and cellular cobamide concentrations were calculated assuming 8 × 10^8^ cells/ml at an OD_600_ of 1.0 and a cellular volume of 1 μm^3^.
