# Supplementary figures and images for: Cofactor Selectivity in Methylmalonyl Coenzyme A Mutase, a Model Cobamide-Dependent Enzyme
Source: mBio. 2019 Sep 24;10(5):e01303-19. doi: 10.1128/mBio.01303-19 (PMC6759758; doi:10.1128/mBio.01303-19)

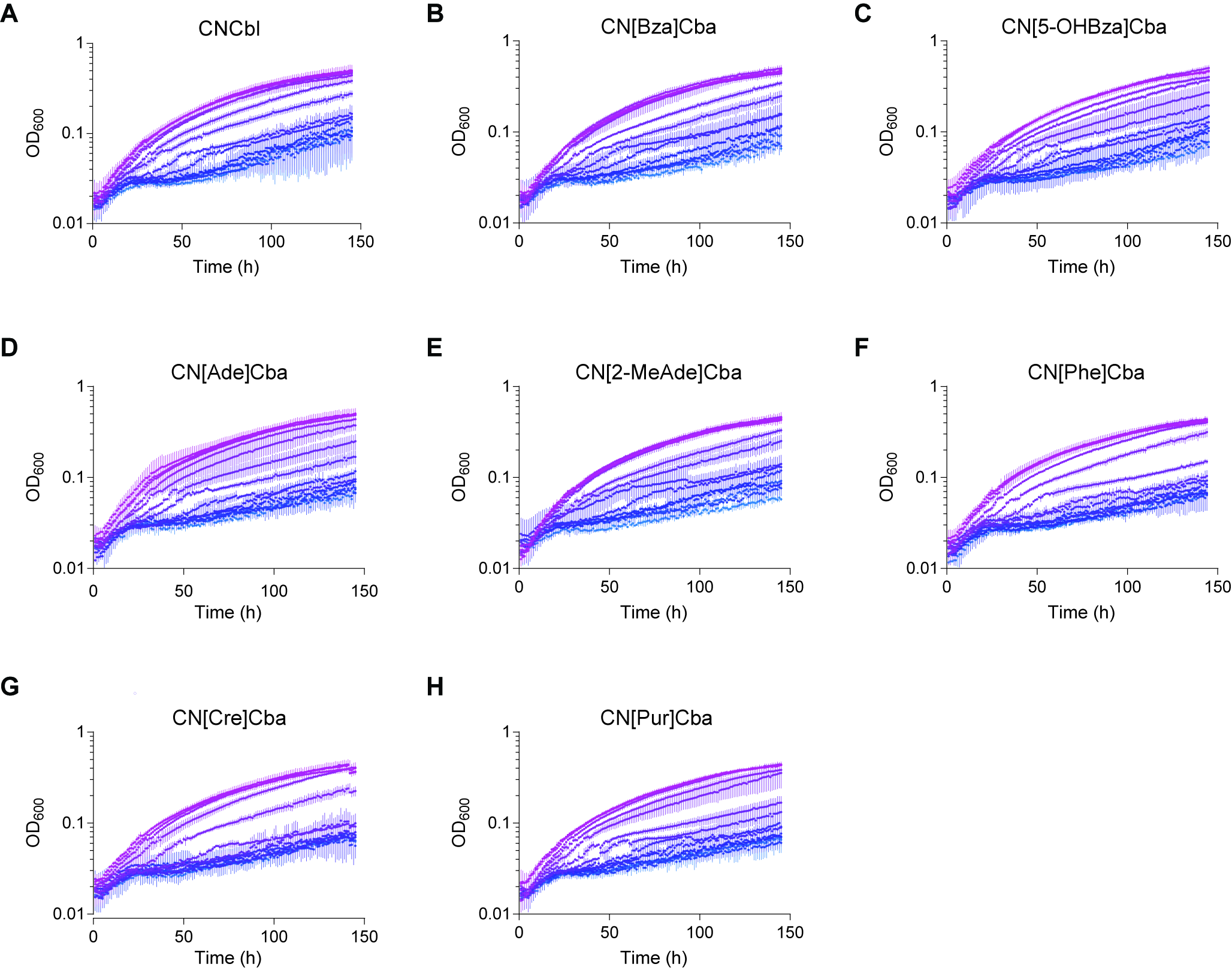

Supplement: FIG S4 [file mBio.01303-19-sf004.tif]

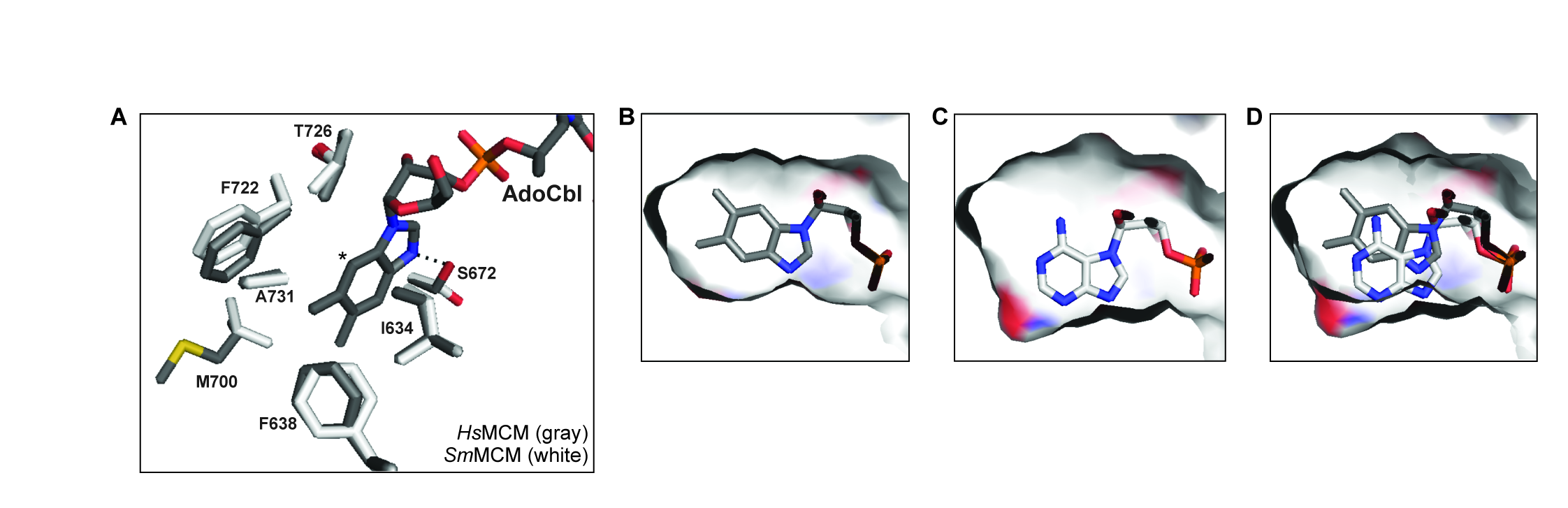

Supplement: FIG S6 [file mBio.01303-19-sf006.tif]

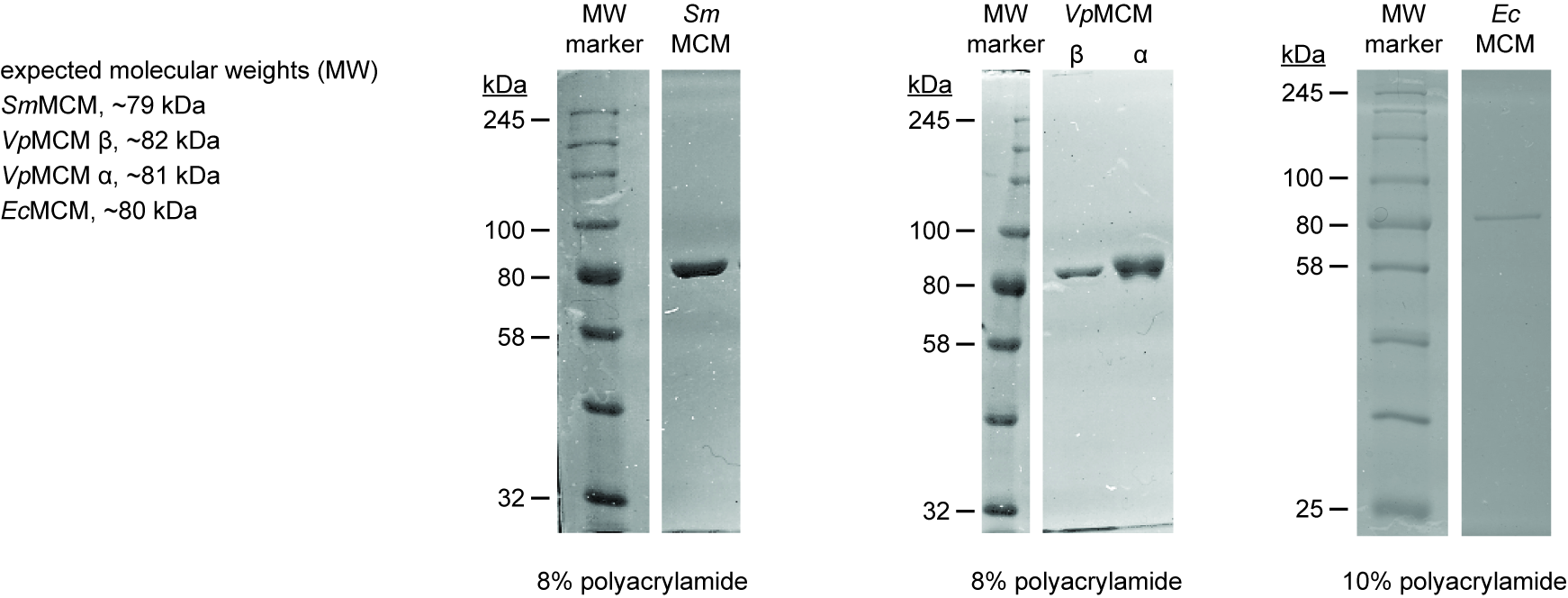

Supplement: FIG S7 [file mBio.01303-19-sf007.tif]
