# Supplementary material for: Cofactor Selectivity in Methylmalonyl Coenzyme A Mutase, a Model Cobamide-Dependent Enzyme
Source: mBio. 2019 Sep 24;10(5):e01303-19. doi: 10.1128/mBio.01303-19 (PMC6759758; doi:10.1128/mBio.01303-19)
Supplement: TABLE S1 [file mBio.01303-19-st001.docx]

| Construct | Primer sequence |
| --- | --- |
| pET28a-*bhbA*  (*Sm*MCM) | ccgcgcggcagccatatggctagcACCGAAAAGACCATCAAGGACTG  aagcttgtcgacggagctcgaattcTTACACGTTTCGCCGCTTGC |
| pET28a-*scpA*  (*Ec*MCM) | cgcgcggcagccatatgATGTCTAACGTGCAGGAGTGG  gtgcggccgcaagcttTTAATCATGATGCTGGCTTATCAG |
| pTH1227-Vpar_RS06295- Vpar_RS06290 | ttcacctcgagatctatcgatgcatACCTGTTTTTTTTAGGAGGATGATGAAAAC gcttgaattcgagctcccgggtaccTTATTTTACGTTTTCTTTAATAAAGTTAACGATATCGC |
| pTH1227-Vpar_RS09005-Vpar_RS09000 | ttcacctcgagatctatcgatgcatGGTTTCGAGTTGAAAAGGAGGCA  gcttgaattcgagctcccgggtaccTCAGTCATGAGACTCGTCTTGAAC |
| pETDuet-Vpar_RS09005 (*Vp*MCM α) | cattggatcctATGTCTGACAAAAAGAC  ctaactcgagTCAGTCATGAGACTCGTC |
| pETDuet- Vpar_RS09000 (*Vp*MCM β) | cattggatcctATGTTTAAAAATC  ctaactcgagTCAGTCATGAG |
