# Supplementary material for: Cofactor Selectivity in Methylmalonyl Coenzyme A Mutase, a Model Cobamide-Dependent Enzyme
Source: mBio. 2019 Sep 24;10(5):e01303-19. doi: 10.1128/mBio.01303-19 (PMC6759758; doi:10.1128/mBio.01303-19)
Supplement: TABLE S2 [file mBio.01303-19-st002.docx]

| Compounds purified | Column | Mobile Phases | Flow (mL/min) | Temp  (˚C) | Gradient |
| --- | --- | --- | --- | --- | --- |
| Phenolyl, non-polar benzimidazolyl, and adenosylated cobamides | b | A: 0.1% formic acid in water  B: 0.1% formic acid in methanol | 2 | 30 | 25% B, 2 min  25 – 60% B, 24 min |
| Purinyl cobamides and azabenzimidazolyl cobamides | b | A: 0.1% formic acid in water  B: 0.1% formic acid in methanol | 1.5 | 15 | 10 – 30% B, 3 min  30% B, 13.9 min  30 – 37% B, 2.1 min |
| Purinyl cobamides and azabenzimidazolyl cobamides | b | A: 0.1% formic acid in water  B: 0.1% formic acid in methanol | 2 | 30 | 10 – 42% B, 20 min |
| [5-OHBza]Cba | b | A: 0.1% formic acid in water  B: 0.1% formic acid in methanol | 2 | 25 | 18 – 25% B, 2.5 min  25%, 22.5 min |
| β-adenosylcobinamide (Widner FJ, 2013, Ph. D. Leopold-Franzens-University Innsbruck) | b | A: 10 mM sodium phosphate pH 7  B: acetonitrile | 2 | 25 | 2 – 23% B, 40 min |
| methylmalonyl-CoA | b | A: 0.1% formic acid in water  B: acetonitrile | 3 | 25 | 0 – 10% B, 30 min  10 – 70% B, 3 min |
| methylmalonyl-CoA (analytical) | a | A: 100 mM acetic acid, 100 mM sodium phosphate, pH 4.6  B: 100 mM acetic acid, 100 mM sodium phosphate, pH 4.6  18% methanol | 0.75 | 40 | 44% B, 14 min |
| Column a: Eclipse XBD-C18, 4.6 x 150 mm, 5µm  Column b: Zorbax Eclipse Plus C18, 9.4 x 250 mm, 5µm | | | | | |
